# Which product characteristics are preferred by Chinese consumers when choosing pork? A conjoint analysis on perceived quality of selected pork attributes

**DOI:** 10.1002/fsn3.457

**Published:** 2017-01-22

**Authors:** Xiu Q. Ma, Julia M. Verkuil, Helene C. Reinbach, Lene Meinert

**Affiliations:** ^1^ Department of Development and PlanningAalborg University CopenhagenCopenhagenDenmark; ^2^ Danish Meat Research InstituteTaastrupDenmark

**Keywords:** China, conjoint analysis, consumer decision‐making, imported, pork, quality attributes

## Abstract

Due to the economic growth achieved by China over the past 20 years, Chinese consumers have changed their purchasing behavior regarding meat. Instead of buying locally produced pork, they are increasingly willing to purchase imported pork. A conjoint analysis investigated how intrinsic pork attributes (*fat content* and *processing)* and extrinsic pork attributes (*origin*,* price*, and *packaging*) relate to the perceived quality of pork and the choices made by Chinese consumers. A questionnaire distributed among a sample of Chinese consumers (*n* = 81) revealed that processing (fresh/frozen) is the most important determinant of pork choice (36%), followed by fat content (27%), origin (18%), price (12%), and packaging (6.6%). Estimates of utility showed that Chinese consumers value fresh pork highly (0.147), followed by lean pork (0.111) and pork imported from countries other than China (0.073). The findings indicate that Chinese consumer's value both intrinsic and extrinsic attributes, and these results may help the meat industry improve China's competitive meat market by developing new and more products that are tailored to the needs of the consumer.

## 1 Introduction

China, with its large population, has achieved remarkable economic growth over the past 20 years and has become a major player in the global economy. In line with these positive economic developments such as increase of income, many related social changes affecting lifestyles, dietary habits, and consumer behavior have taken place (Huang & Yang, 2009). One of the consequences of these transitions is a strong expansion of the Western lifestyle, which has led to an increase in the consumption of animal products (e.g., pork and beef), and this trend is continuing to grow (Nam, Jo, & Lee, 2010). In China, pork has been the dominant animal protein source in the traditional Chinese diet and now accounts for more than 64% of the meat consumption in the country (Verbeke & Liu, 2014) (EU SME Centre, 2013).

With an increasing demand for pork, local meat producers in China are unable to meet the needs of their consumers, thereby creating both export opportunities and an increased consumption of imported pork. Furthermore, frequent food safety incidents in China have received considerable attention in recent years, in particular, the melamine‐contaminated milk scandal of 2008 (Knight, Gao, Garret, & Deans, 2008). For this reason, many Chinese consumers prefer to buy imported pork rather than locally produced pork and do not only consider the price of the meat but also increasingly factors such as safety, quality, and health (Henchion, McCarthy, Resconi, & Trov, 2014). This gives imported food products a better reputation, resulting in an increased demand for these products in China. Liu, Pieniak, and Verbeke (2013) found that Chinese consumers demonstrate a high degree of willingness to pay more for safe, high‐quality food. This increase in demand, coupled with the fact that Chinese consumers are interested in accessing a wider range of safe, high‐quality products makes mainland China a potential and important consumer market for food producers worldwide (Zhang, 1996).

There are only a few studies investigating pork consumption in China that relate the quality of pork to its product attributes. Grunert, Loose, Zhou, and Tinggaard (2015) conducted a choice‐based conjoint analysis and showed that intrinsic cues (fat and color) influence the choice of pork and found that Chinese consumers consider freshness to be an important quality attribute. Several studies have shown that the origin of the meat has an impact on the purchase of meat (Acebron & Dopico, 2000) (Banovic, Grunert, Barreira, & Fontes, 2010) (Hoffmann, 2000), although none of them were specifically focused on pork or based on the Chinese market. However, the country of origin has previously been found to have an impact on the overall product evaluation and the perception of food (Yeh, Chen, & Sher, 2010).

Therefore, the aim of this study was to investigate Chinese consumer responses to selected pork attributes in order to understand how these characteristics relate to the perceived quality and choice of pork. More specifically, a conjoint analysis is used to investigate how Chinese consumers value a combination of product attributes when choosing pork: *price* (expensive and cheap), *origin* (meat produced locally in China and meat imported from other countries), *fat content* (lean and fat), *packaging* (skin: product on paperboard with cover of thin transparent plastic, no air and vacuum: product in plastic bag, no air), and *processing* (fresh and frozen).

## 2 Material and Methods

### 2.1 Questionnaire development

A questionnaire was developed to investigate how Chinese consumer choices of selected pork attributes relate to the perceived quality and choice of pork. It included demographic measures (*gender, age, marital status, education level, family size, income level, residence)*, a screening question to assess the consumption of locally produced and imported pork, and also a section with conjoint questions to evaluate the relative importance of selected pork attributes for Chinese consumers when choosing pork.

**Figure 1:**
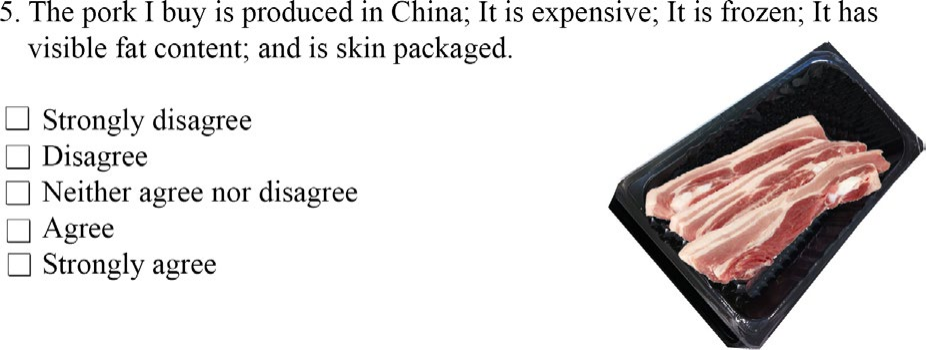
An example of a graphical product simulation from the conjoint questions using the 5‐point Likert scale

The conjoint analysis included both intrinsic and extrinsic factors (see Table 1) visualized by graphical product simulations (see Figure 1). Since pictures are easy for the respondents to understand, they are close to real‐world choices, and visual components have been found suitable for measuring preferences for product attributes such as packaging and visual marbling/fat content (Grunert et al., 2015) that relate to the perceived quality of pork.

**Table 1:** Intrinsic and extrinsic attributes and their levels of perceived quality

Attributes	Origin	Price	Process	Fat content	Package
Levels	1. Locally produced in China	1. Expensive	1. Frozen	1. Fat	1. Skin packaging
	2. Imported from other countries	2. Cheap	2. Fresh	2. Lean	2. Vacuum packaging

A nonorthogonal but optimal design was developed for the conjoint analysis with a total of 16 choice sets derived from the selected characteristics (*origin, price, processing, fat content, and packaging*), including four holdouts. The conjoint questions were measured using a 5‐point Likert scale (*1: “strongly disagree”, 5: “strongly agree”*), including a neutral option in order to make the respondents express their opinion without any pressure. The following is an example of a choice: *“The pork I buy is produced in China; it is expensive; it is frozen; it has visible fat content and is skin packed”* (see Figure 1).

The questionnaire was developed in English and subsequently translated into Chinese. The questionnaire was then pretested to Chinese students in Denmark, leading to minor modifications of the grammatical structure of the questions and the addition of the screening question to assess the consumption of locally produced and imported pork.

### 2.2 Subjects and data collection

A convenience sample (*n* = 507) of Chinese people living in mainland China were recruited via social media (e.g., Wehat, QQ, and Microblog) and were asked to complete a self‐administered questionnaire comprising 16 questions, which included a short text that guided the participants when completing the questionnaire.

Participants who did not consume either locally produced or imported pork were excluded from the study. A significant proportion of the participants did not consume imported pork (*N* = 199) and were excluded. Approximately 200 participants only partially completed the questionnaire because of insufficient loading of questions and images due to insufficient Internet connection, and they were also excluded from the study.

Data collection was initiated on 27 February 2015, which is the first workday after the Chinese New Year “Chun Jie” holiday. The participants had until 15 March 2015 to respond. The final sample consisted of 81 participants who consumed both locally produced and imported pork.

### 2.3 Analysis

Socio‐demographics are presented as percentages and frequencies. The conjoint analysis was performed using SPSS 22.0 (SPSS statistics for Mac, Version 22.0, Armonk, NY, IBM Corp.) to estimate relative importance utilities (IBM, 2005). The conjoint analysis and utilities were applied in this study to identify the relative importance of selected pork attributes for Chinese consumers when choosing pork meat. The Pearson's and Kendall tests were used to test the validation of the model.

## 3 Results

### 3.1 Socio‐demographics

The socio‐demographic characteristics of the participants (*N* = 81) are presented in Table 2. Female respondents accounted for 56% of the participants. Most of the respondents were aged 30–39 years (51%), followed by the age group 20–29 years (22%). The majority of the respondents were married/lived with a partner and had children (65%). A large proportion of the participants (52%) lived in a three‐person household, and only a small proportion (1%) lived alone. Approximately, one‐third had a medium‐high income (5,001‐10,000 RBM/month, 1 RBM = 0.14 Euro), while only 3% belonged to the low‐income group (<1,500 RBM/month).

**Table 2:** Socio‐demographics of Chinese consumers (*N* = 81)

Variable	*N*	%
Gender		
Male	36	44
Female	45	56
Age		
<19 year	1	1
20–29 year	18	22
30–39 year	41	51
40–49 year	17	21
50–59 year	4	5
60–69 year	0	0
>70 year	0	0
Marital status		
Single	14	17
Married/partner without children	11	14
Married/partner with children	53	65
Divorced/widowed with children	3	4
Divorced/widowed without children	0	0
Family size		
1 person	1	1
2 persons	14	17
3 persons	42	52
4 or more persons	24	30
Income/month (RBM)		
<1,500	2	3
1,500–3,500	14	17
3,501–5,000	23	28
5,001–10,000	25	31
10,001–150,000	6	7
<150,001	11	14
Education		
Primary school	0	0
Middle school	7	9
Technical secondary	5	6
Senior school	6	7
Diploma	12	15
Bachelor	33	41
Master	14	17
PhD	4	5
Resident		
Municipality (Beijing, Shanghai, Tianjin, Chongqing)	9	11
Province capital	13	16
Big city (population 1–5 million)	33	41
Medium city (population 0.1–1 million)	14	17
Small city (population 0.2–0.5 million)	10	12
Town or country village	2	3

The majority (63%) of the respondents in this study had a high education level, which included 41% bachelor's, 17% master's, and 5% PhD. Respondents in this study were based mainly in big cities (41%), while a low number of respondents were based in towns or country villages (3%).

### 3.2 The relative importance of selected pork attributes

The estimated utilities from the conjoint analysis (Table 3) suggested that processing was the most important driver for the choice of pork, accounting for 36% of the variance in the dataset. Fat level was the second most important pork attribute (27%), followed by origin (18%), price (12%), and packaging (6.6%). Packaging had a very low variance share, indicating that it was the least important value in this study. Specifically, Chinese consumers valued pork imported from other countries that was expensive, fresh, lean, and that came with skin packaging.

**Table 3:** Conjoint analysis results for quality attributes

Factors	Level	Utility	Importance values %
Origin	Imported from other countries	0.073	18.033
	Locally produced in China	−0.073	
Price	Expensive	0.049	12.022
	Cheap	−0.049	
Process	Frozen	−0.147	36.066
	Fresh	0.147	
Fat content	Fat	−0.111	27.322
	Lean	0.111	
Package	Skin	0.027	6.557
	Vacuum	−0.027	
(Constant)		3.129	
Pearson's R		0.885	
Kendall's tau		0.718	
Kendall's’ tau for holdouts		0.183	

The conjoint analysis (see Table 3) showed that the Pearson's R and Kendall's tau were high and significant, indicating that the model fitted the data and could describe the importance of the selected pork attributes well (Pearson's R = 0.885; Kendall's tau = 0.718, *p* < .001).

## 4 Discussion

In this study, Chinese consumer responses to pork characteristics (e.g., *price*,* origin*,* fat content*,* processing*, and *packaging*) were investigated to explore the extent to which these selected attributes influence the choice of pork. The overall findings show that attributes such as *fresh*,* lean*,* imported from countries other than China*,* expensive*, and *skin packaged* were highly valued. Chinese consumers therefore focus on both intrinsic attributes (e.g., *fat content* and *freshness*) and extrinsic attributes (e.g., *origin* and *packaging*) when choosing pork. In agreement with previous studies, this supports the fact that intrinsic attributes (*fat content* and *processing*) are important drivers of choice and are highly related to perceived pork quality (Verbeke & Liu, 2014) (Furnols & Guerrero, 2014) and that extrinsic quality attributes (*packaging*,* price*, and *origin*) can support the evaluation of quality.

Of the selected pork attributes in this study, fresh pork is given the highest relative importance, which might be related to the fact that Chinese consumers are used to buying fresh pork at outdoor farmers’ markets. A study by Zhou, Zhang, and Xu (2012) showed that fresh meat is still the predominant type of meat sold in rural areas. This result is in agreement with a study by Grunert et al. (2011), who reported that Chinese consumers placed the greatest importance on freshness as a quality attribute for meat. However, today the trend in meat consumption patterns in China has changed from fresh (non‐chilled) to frozen and, more recently, from frozen to chilled fresh meat, especially in big cities like Beijing and Shanghai (Zhou et al., 2012). However, in most parts of China, especially in rural areas, farmers’ markets are still the predominant sellers of fresh meat. To the Chinese, “fresh” does not merely mean “not frozen or canned” but, more precisely, food that comes directly from the farm (Grunert et al., 2011).

In this study, lean pork is valued more highly than fatty pork, indicating a higher perceived quality of lean meat, which might be linked to consumers’ health and nutritional concerns. Sanders, Moon, and Kuethe (2007) reported similar results and pointed out that consumers are more likely to buy fresh and lean pork based on recent health concerns. Furthermore, consumers have previously reported that fatty pork has a bad taste and therefore has a lower overall perceived quality (Zhou et al., 2012). The fact that health and nutritional concerns are increasingly a priority for Chinese consumers (Liu et al., 2013) could be of interest to the meat industry, e.g. in future focus on lean cuts.

Interestingly, the current results show that perceived high‐quality pork is related to pork imported from countries other than China as opposed to locally produced pork. Likewise, Zhou et al. (2012) showed that Chinese consumers are willing to pay more for imported pork due to the good reputation of imported food products. This indicates that Chinese consumers perceive food products from other countries as high quality (Yeh et al., 2010) (Knight et al., 2008). For example, products originating from developed countries tend to receive a higher evaluation than those from undeveloped countries (Yeh et al., 2010). Another reason for the good reputation of imported food products is the high number of incidents concerning unsafe domestic products in China (Knight et al., 2008).

Especially participants with a medium‐high income reported buying both imported and locally produced pork (59%, see Table 2). Furthermore, expensive pork was valued highly by the Chinese consumers, indicating that price could be a quality indicator that significantly influences their choice of pork. Likewise, an earlier study by Henchion et al. (2014) showed that price can influence consumers’ choice of pork in Asia. In China, the price of imported pork is higher than the price of locally produced pork, and this might explain why, in particular, consumers with medium to high incomes are willing to pay the higher price for imported pork. Besides the economic situation, individual concerns, such as health and nutrition, play an important role in consumer behavior (Henchion et al., 2014). In this study, most of the participants were part of a three‐person household, reflecting the traditional one‐child policy in China. Children play an important role in the financial and caretaking responsibilities of families in China, and more Chinese people prefer to purchase imported food products for their children because they feel that those products are safer (Veeck & Burns, 2005). Furthermore, Chinese consumers are willing to pay more for safe and high‐quality food products, for example, imported food products, since these products are generally considered to be of better quality than locally produced food products (Zhou et al., 2012).

Skin packaging received a better rating than vacuum packaging, although packaging has the lowest relative importance value compared to the other attributes (*price*,* origin*,* fat content*, and *processing*). This could indicate that, for Chinese consumers, packaging is not an important or well‐known attribute when it comes to perceived quality of pork. The reason for this could be that, traditionally in China, consumers buy pork at outdoor farmers’ markets, where the meat is cut directly from the carcass according to the consumers’ needs (e.g., fat content and weight). Therefore, many consumers still prefer to buy pork that can be both seen and touched. In the supermarket, packaged pork cannot be touched and has already been cut into pieces, leaving the consumer with limited choices. Furthermore, lack of experience and knowledge of different packaging types might explain why packaging is of little importance to Chinese consumers.

From a methodological perspective, further research could be conducted with a larger sample size representative of Chinese consumers to evaluate pork attributes related to perceived quality. For example, Chinese three‐person households (reflecting the previous one‐child policy) with a medium income could be targeted, since children play a significant role in the financial responsibilities of families and since nowadays Chinese people prefer to purchase safe products (e.g., imported products) for their children (Veeck & Burns, 2005). The graphical pictures and accompanying text in the conjoint analysis were found useful in evaluating the importance of different pork attributes on choice of pork and in relating these to perceived quality. However, only two levels of each attribute were included (e.g., *cheap/expensive*), and some attributes might be unfamiliar to the consumers (e.g., *skin* and *vacuum packaging*), which may have influenced the evaluation of pork quality. In addition, the questionnaire was pilot‐tested with Chinese students in Denmark. It would have been optimal for the study design if the pilot test had also focused on the target group of this study. Furthermore, the text used in the questionnaire to describe the figures (see Figure 1) is relatively complex and contains a large amount of information, and this might have led to differences in understanding the questions. Another drawback of the methodology was that participants needed access to a computer to complete the questionnaire and sufficient Internet speed to load the images attached to the questions. In China, many rural households were unable to access computers or did not have access to a fast Internet connection. In this study, many of the households did not have access to a fast Internet connection, which meant that questions and images could not be loaded. This explains why approximately 200 participants only partially completed the questionnaires, and these could not be used in this research.

Despite these limitations, the study illustrates how Chinese consumers evaluate pork attributes that are important for perceived quality. These findings, in combination with recent literature, indicate a potential for addressing the importance of selected attributes to improve quality attributes of pork (*packaging*,* fat content*, and *processing*) in the meat industry. The findings also highlight the need to adopt a relevant food safety policy to guarantee and maintain meat safety and ensure that safe, quality products are sold to consumers in China.

## 5 Conclusion

It can be concluded that the attributes *fresh*,* lean*,* imported from countries other than China*,* expensive*, and *skin packaged* are highly valued by Chinese consumers when choosing pork and evaluating pork quality. Both intrinsic and extrinsic attributes influence the perception of pork quality, and therefore a better understanding of these attributes may help the meat industry improve China's competitive meat market. For instance, the perceived quality of pork can be enhanced using effective strategies of communication targeting different groups (e.g., young people, families, and children) and using labels on pork products that are recognized by the consumer.

## Conflict of Interest

None declared.
